# Inclusiveness of Access Policies to Maternity Care for Migrant Women Across Europe: A Policy Review

**DOI:** 10.1007/s10995-023-03785-3

**Published:** 2023-10-16

**Authors:** Alena Pařízková, Jette Aaroe Clausen, Marie-Clare Balaam, Melanie Haith-Cooper, Triin Roosalu, Laura Migliorini, Anne Kasper

**Affiliations:** 1 Department of Sociology and social work, Faculty of Arts, University of West Bohemia, Univerzitní 8, Plzeň, 30100 Czech Republic; 2 Consultant in the Taenk og tal, Blågårdsgade 28, Copenhagen, 2200 Denmark; 3 Research in Childbirth and Health Unit (REACH), School of Nursing and Midwifery., University of Central Lancashire, Preston, PR1 2HE 01772 893885 UK; 4 Faculty of Health Studies, University of Bradford, Richmond Road, Bradford, West Yorkshire UK; 5 School of Governance, Law and Society, Institute of International and Social Studies, Tallinn University, Uus-Sadama 5, Tallinn, 10120 Estonia; 6 Department of Education Sciences, University of Genoa, C.so Podestà 2, Genoa, 16156 Italy; 7 Health Campus Göttingen - Faculty of Engineering and Health, HAWK University in Hildesheim, Holzminden and Göttingen, 31134 Hohnsen 4, Hildesheim, Germany

**Keywords:** Migrant women, Maternal health care access, Structural inequalities, European policies, Inclusion

## Abstract

**Introduction:**

Despite the interconnectedness of the European Union, there are significant variations in pregnant women’s legal status as migrants and therefore their ability to access maternity care. Limited access to maternity care can lead to higher morbidity and mortality rates in migrant women and their babies. This study aimed to investigate and compare maternal health access policies and the context in which they operate across European countries for women who have migrated and are not considered citizens of the host country.

**Methods:**

The study adopted a mixed-methods research design exploring policies on migrant women’s access to maternity care across the migration regimes. Data were extracted from legal documents and research reports to construct a new typology to identify the inclusiveness of policies determining access to maternity care for migrant women.

**Results:**

This study found inconsistency in the categorisation of migrants across countries and significant disparities in access to maternity care for migrant women within and between European countries. A lack of connection between access policies and migration regimes, along with a lack of fit between policies and public support for migration suggests a low level of path dependency and leaves space for policy innovation.

**Discussion:**

Inequities and inconsistencies in policies across European countries affect non-citizen migrant women’s access to maternity care. These policies act to reproduce structural inequalities which compromise the health of vulnerable women and newborns in reception countries. There is an urgent need to address this inequity, which discriminates against these already marginalised women.

## Significance

Some migrant women in Europe struggle to access maternity care when compared to non-migrant women, resulting in poorer pregnancy outcomes. Systematic inconsistencies in maternal health care access policies across Europe reveal the extent of inequity faced by vulnerable migrant women. Our analysis demonstrated significant policy differences across and within migration regimes, but also highlighted that some countries do not have a good fit between policies regulating migrants’ access to maternity care and public or policy level support for migrants. This points to limited relevance of path dependency in these policies, suggesting that a more inclusive approach across Europe might be possible.

## Introduction

Some migrant women struggle to access optimal maternity care in their host country and experience poorer pregnancy outcomes than non-migrant women (Heslehurst et al., 2018; Gieles et al., 2019; Fair et al., 2020). Migrants’ right to health care is closely linked to their legal status within their host country (World Health Organisation [WHO], 2008). Despite the interconnectedness of the European Union, there are significant variations in pregnant women’s legal status as migrants in different countries and therefore their ability to access optimal maternity care (Rechel et al., 2013; Women Political Leaders [WPL], 2017). Women make up more than half of the migrating population and large numbers of these are women of childbearing age (WHO, 2018b), therefore it is essential that these variations are challenged at a European level to ensure equity of care.

A concept analysis identified ambiguity around the term pregnant migrant woman and the importance of considering the heterogeneity of migrant women in policy, practice, and research (Balaam et al., 2017). Some groups of migrants, particularly those identified as forced migrants or displaced persons, (who include asylum seekers, refugees, trafficked individuals and those who have no documentation) may have a more precarious and less stable legal status in their host country leading to difficulties accessing health care (Bollini et al., 2009; Gieles et al., 2019; WPL, 2017). They are also more likely to experience poorer underlying physical and mental health, with high levels of socioeconomic and financial disadvantage in their host country (WHO, 2018a). Pregnancy and motherhood commonly exacerbate poor health, poverty, and deprivation (Jones et al., 2022) with the stress of migration adding to the challenges linked to the transition to motherhood (Afflerback et al., 2014; Coutinho et al., 2014). A poor experience at this time can influence the health in later life of both woman and infant (Redshaw et al., 2019). Consequently, the reproductive health of migrant women is of increasing concern to researchers, practitioners, and policymakers (WPL, 2017; WHO, 2018a).

A country’s maternity care policy for pregnant migrant women can substantially influence the health of women and babies. Limited access and poor-quality maternity care can lead to higher morbidity and mortality rates for some migrant women (Bollini et al., 2009; Fair et al., 2020). In countries with existing restrictive legal and bureaucratic structures, maternity care providers can find themselves facing challenges especially when meeting the needs of some newly arrived migrants (Boerleider et al., 2013; Suurmond et al., 2013; Letley, 2022). European countries vary in the adherence to WHO Standards for maternal care (Iannuzzi et al., 2018) and it is important to explore and compare the maternal care policies of different European country in order to challenge differences in care provision and improve maternal health.

A systematic literature review found no studies comparing maternal care access policies in different European countries. We did however find studies addressing health policy in general (e.g. Mladovsky et al., 2012). In addition, several studies have identified a link between (im)migration policies and public opinion, with more negative attitudes towards migration leading to more restrictive (im)migration policies (Morales et al., 2015) if migration is a salient issue (Bohmelt, 2021). This link has not been explored in the context of health, and more specifically maternal care access policies and creating positive public attitudes towards migration could influence maternal care policy makers.

The WHO and WPL have called for more coordinated initiatives across Europe to address inconsistencies in access to maternity care for migrant women. This includes the need for quality research and data collection to improve the evidence base and support policy change (WPL, 2017; WHO, 2018a). It is important to understand the variations in maternity care access policies between different countries so that these variations can be challenged at a European level.

Our study aimed to investigate and compare the maternal care access policies across European countries for women who have migrated and are not considered citizens of the host country. We asked to what extent do migrant women have access to care that is comparable to standard maternity care in their host country? To illustrate the context in which maternal health policies operate within different countries, we also developed a typology to determine how inclusive or exclusive the policies of these countries are towards migrants. We then explored the connection between the national context and the level of access to standard maternity care within a country.

## Methods

The study used a mixed-methods approach to collect secondary data. We identified 14 different European countries to explore as case studies. These were selected because they represented the variability of countries in relation to migration. First, we analysed data related to access to maternity care using qualitative thematic analysis. Then, we created a typology containing indicators representing factors that interact with government policy. The last step was to develop indexes.

### Exploring Migrant Women’s Access to Maternity Care

As researchers we worked within an EU funded international network of experts in maternal healthcare and related fields. Information on national health care policies for migrants was collected by these experts extracting data from current publicly available and credible sources developed by Government Authorities and research institutions across Europe and translated this into English. Information was requested, for documents focusing on two areas: (i) existing categories and definitions of non-citizens in the country and related rules influencing access to health care, (ii) opportunities for and limitations of access to maternity care for non-citizens.

We developed a template to standardise the responses and the experts from the international network completed the template for fourteen countries in 2016, with updates in late 2017 and in 2018 to ensure changes in policy were captured. To strengthen the validity of the data and limit the risk of bias, we made the three-step data collection and revision process. For each country, in the first step one expert collected data, secondly another expert revised the data and finally two members of the research team revised the data and checked the sources from all countries. All the experts (as authors) were members of the international expert network COST Action IS1405 BIRTH. Their membership was based on their expert status and approved by the Action’s lead. The extracted data were then subject to thematic analysis (Braun & Clark, 2006). This process was undertaken by two members of the research team to enhance validity. We benchmarked our findings against the care available to women registered as citizens in each country or care that is considered ‘standard care’. We identified three major themes: legal and residency status as a dividing line among all non-citizens; conditions of access; types of care. Furthermore, we identified two categories relating to the access policies settings: (1) we describe policy as ‘inclusive’, if pregnancy was the only precondition for migrant women to access standard maternity care in the given country, (2) and ‘limited’, if there were additional conditions applied (see Table 1).

**Table 1:** Factors influencing access to maternity care in different countries

Country	Recognition of pregnancy as sufficient condition of providing access to standard maternity care	Additional factors for accessing maternity care services	Inclusive or limited access policy
Croatia	yes	none	Inclusive
Cyprus	no	Certificate	Limited
Czech Republic	no	Legal status, insurance, employment status	Limited
Denmark	no	Residency, legal status	Limited
Germany	yes	Legal status, employment status, insurance	Limited
Greece	no	Country of origin, type of residency, legal status, economic insecurity	Limited
Hungary	no	Residency, legal status	Limited
Italy	yes	none	Inclusive
Ireland	no	Residency, own resources	Limited
Netherlands	no	Insurance, legal status	Limited
Norway	yes	none	Inclusive
Malta	yes	none	Inclusive
Spain	yes	none	Inclusive
Switzerland	yes	none	Inclusive
United Kingdom	yes	none	Inclusive

Source: own analysis

### Building the Typology of Country Contexts

Drawing on techniques from qualitative comparative analysis and its case-orientated approach (Weis & Willems, 2017), we interpreted each country as a case study that we described using indicators that we pre-selected. These characterised a country’s policy in terms of migration experience and access to maternity care. We focused on these groups of indicators:

1. Country context: we selected data which characterised the economic position of the country, geography, migration experience and type of immigration controls (Boucher & Gest, 2015) (Table 2).
  **Table 2:** Typology of migration regime and country context

Type	Migration regime		Country	Location in migration context	EU accession	GDP (millions US)^1^	Subjective positive judgment of the situation of		Number of migrants in the country (thousands)^3^	Share of migrants in total population (%)^3^
A	Southern border countries	Southern Islands	Cyprus	Border island	2004	20,047.01			189	16
			Malta	Border island	2004	10,999.05	79	62	46	11
		New countries for immigration	Italy	Border country	1958	1,859,383.61	63	54	5,907	10
			Spain	Border country	1986	1,859,383.61	60	56	5,947	13
			Greece	Border country	1981	192,690.81	35	30	1,220	11
B	Post-colonial and guest workers system		UK	Target country	1973	2,650,850.18	80	72	8,842	13
			Germany	Target country	1958	3,477,796.27	82	70	12,165	15
			Netherlands	Target country	1958	777,227.54	91	65	2,057	12
			Denmark	Target country	1973	306,899.65	92	78	657	12
			Norway	Target country	No	371,075.24			799	15
			Switzerland	Target country	No	668,851.30			2,506	30
C	Rejecting countries		Croatia	Transfer / CEE	2013	50,714.96	48	48	560	13
			Hungary	Transfer / CEE	2004	125,816.64	56	55	504	5
			Czech Republic	CEE	2004	195,305.08	75	63	433	4
	Note			CEE - Central and East Europe			How would you judge current situation in each of the following? (%) (1) Financial situation of your household. (2) Your personal job situation. We show percentage of “Total ‘good’” selection.			

Sources:^1^ World Bank, n.d., ^2^ European Commission, 2018a, ^3,^ UN, 2017
2. Public support towards migration, migrants and their inclusion in health care: we reviewed surveys and statistics in documents from the World Bank, European Commission and ISSP Research Group, to describe the countries by their migration regimes and selected indicators illustrating their approach to migrants (Table 3).
  **Table 3:** Migration regime and indicators on public support towards migration, migrants and their inclusion in health care

Type	Migration regime		Countries	Perceived importance of migration as a national issue and for EU^1^	Support for common EU policy on migration^1^	migration perceived as a problem^1^	Migrant Acceptance^3^	Acceptance for public healthcare to be extended to non-citizens^4^
				1…2	1…3	1…4	1…3	1…3
A	Southern border countries	Southern Islands	Cyprus			3	2	
			Malta	1	2	4	2	
		New countries of migration	Italy	1	2	3	1	3
			Spain	2	1	1	1	2
			Greece	2	2	4	2	
B	Post-colonial and guest workers system		UK	2	2	2	1	1
			Germany	1	1	2	1	3
			Netherlands	2	1	2	1	3
			Denmark	1	2	1	1	3
			Norway			1^2^	1	2
			Switzerland			1^2^	1	3
C	Rejecting countries		Croatia	2	2	2	3	3
			Hungary	1	3	4	3	
			Czech Republic	2	3	3	3	1
		Notes	Value	How important is migration for the EU; for the country 1) equally important both in EU and in country 2) more important for EU than country	Please tell me whether you are for it or against it: A common European policy on migration. 1) > 75% support, less than 20% against (consensus) 2) 50…75% support, 21–49% against 3) < 50% support, about 50% against	EC2018: Do you think migration from outside the EU is more of a problem or more of an opportunity for (COUNTRY) today? We looked at answers: Immigration is more of a problem. ESS (2016) whether country is made a better or worse place to live in as a result of migration. 1) good - < 30% 2) average good- 30–45% 3) average bad − 45–60% 4) bad 60 <	0 (least acceptance) = 9.0 (most acceptance) 1) 9 − 6 2) 4–6 3) 0–4	People should have access to publicly funded healthcare even if their do not hold the citizenship in your country? % of agreement 1) 40 2) 26–39 3) > 25

Sources: ^1^ European Commission, 2018b. ^2^ European Social Survey, 2016. ^3^ Esipova et al., 2017. ^4^ ISSP Research Group, 2015
3. Access to health: we searched public sources to obtain details indicating policies on migrants’ access to general health care including data on anti-discrimination and integration strategies (Table 4).
  **Table 4:** Migration regime and indicators on access to health care

Type	Migration regime		Country	Existence of non-discrimination law in the healthcare context^1^				Integration strategy^2^	Additional measures to enable access to care^2^		Policy towards undocumented migrants or migrants in irregular situation			
				Nationality mentioned in anti-discrimination law	Protection of non-nationals in anti-discrimination law	Positive action allowed	Ratification of international anti-discrimination protection	Integration strategy targeting health	Standards on cultural mediators	Free interpreters	Coverage for undocumented migrants^3^	Emergency healthcare^4^	Primary healthcare^4^	Secondary healthcare^4^
				1	1…3	1…2	1…3	1…2	1…2	1…2	1…3	1…3	1…3	1…3
A	Southern border countries	Southern Islands	Cyprus	1	1	2	1	2	2	1	3	3	2	3
			Malta	1	2	2	2	2	1	2	3	2	2	2
		New countries of immigration	Italy	1	2	1	2	1	1	1	2	1	2	1
			Spain	1	1	2	2	1	2	1	1	1	3	3
			Greece	1	3	2	3	2	2	2	3	2	3	3
B	Post-colonial and guest workers system		UK	1	1	1	3	2	2	1	2	1	1	1
			Germany	1	3	2	3	1	1	1	3	1	3	3
			Netherlands	1	1	1	1	2	1	2	1	1	1	1
			Denmark	1	2	2	3	1	1	1	3	1	3	2
			Norway	1	2	2	2				2			
			Switzerland								1			
C	Rejecting countries		Croatia	1	2	1	2	1	2	2		3	3	3
			Hungary	1	1	2	2	2	2	2	3	3	3	3
			Czech Republic	1	2	2	3	2	1	2	3	3	3	3
		Notes	Value	1) yes	1) yes 2) not regulated by law, but in fact provided (yes) 3) no	1) yes + specification 2) no	1) all ratified 2) 1 not ratified 3) 2 not ratified	1) yes 2) no	1) yes 2) no	1) yes 2) no	1) full access 2) greater access 3) only to emergency services	1) for free 2) free with restrictions 3) against payment	1) free 2) with restriction, unclear 3) against payment	1) free 2) with restriction, unclear 3) against payment

Sources: ^1^European Commission, 2017. ^2^ European Website on Integration, 2018. ^3^ Gy, van Ginneken, 2012. ^4^ FRA (European Union Agency for Fundamental Rights), 2015

The origin of the documents was assessed to ensure face validity, relevance to the research aim, authenticity and reliability. Relevant documents were then analysed by two researchers and relevant data extracted and added to an Excel database. To allow better comparability across different indicators, within the surveys and statistics, indexes were developed based on indicators reflected in Tables 3 and 4, following a four-step procedure described by Babbie (2007). Variables were selected which related to our objectives, empirical relationships were examined, then index scoring and index validation took place. Triangulation was applied during all stages of analysis to ensure objectivity and rigour.

We applied an ‘inclusive - limited’ scale as the main analytical frame when developing the indexes to identify the degrees of inclusiveness of selected country policies related to migration and maternal care. Each of these variables was measured in the scale 0–1, with 0 being the most inclusive towards migrants. The higher the score in the index, the less likely a pregnant non-citizen is to receive standard maternity care without additional requirements being met. When scoring ordinal measures of variables, we first identified the average of the values and followed the grades inherited in the values. We then made a composite measure out of several items (details in Tables 3 and 4 in Notes). The resulting indexes are presented in Table 5, ranging from 0.33 to 0.93. Following discussion by the research team a country with a final index value of less than or equal to 0.75 was considered to be inclusive, whereas an index with a value of more than 0.76 was considered by authors of the paper to represent countries with limited maternity care access for non-citizen migrant women.

**Table 5:** Type of migration regime and migrant health care policy support index totals

Migration Regime		Country	Public support towards migration and migrants’ access to health care	Policy towards migrant healthcare	Index score	Inclusive or limited approach to migration
Southern border countries	Southern Islands	Cyprus	0.71	0.81	**0.76**	Limited
		Malta	0.71	0.81	**0.76**	Limited
	New countries of immigration	Italy	0.52	0.57	0.54	Inclusive
		Spain	**0.52**	**0.69**	0.61	Inclusive
		Greece	**0.83**	**0.97**	**0.90**	Limited
Post-colonial and guest workers system		UK	0.70	0.65	0.68	Inclusive
		Germany	**0.40**	**0.78**	0.59	Inclusive
		Netherlands	0.50	0.57	0.53	Inclusive
		Denmark	**0.42**	**0.72**	0.57	Inclusive
		Norway	**0.50**	**0.75**	0.63	Inclusive
		Switzerland	0.39	0.33	0.36	Inclusive
Rejecting countries		Croatia	**0.70**	**0.85**	0.77	Limited
		Hungary	0.88	0.92	0.90	Limited
		Czech Republic	0.95	0.92	0.93	Limited

Source: own analysis based on Tables 3, 4 and 1, and Table 2

This typology of countries describes the national contexts in which the policies determining access to maternity care for non-citizen women were developed and enacted. In the process, we reviewed existing typologies of immigration policies (Cornelius & Tsuda, 2004) and the developments since the “refugee crisis” of 2015 (UNHCR, 2015). Our typology supports the classification of healthcare systems created by Ferreira et al. (2018).

## Results

### Access to Receiving Country’s Standard Maternity Care

We found two principles consistent across all selected countries: the importance of refugee status^1^ and residency, and the definition of childbirth as a condition for urgent medical treatment. Before proceeding to explore the differences, we first discuss the similarities.

Only refugees and those who have gained residency in each country had a clear and consistent definition of their rights to access health care. This was equivalent to citizens of the countries. However, countries commonly defined certain conditions as necessitating emergency medical treatment and these conditions allowed non-citizens to access healthcare. Birth is commonly defined as such a condition and all countries in the study allow all categories of migrant women access to intrapartum care on this basis. In some countries these maternity services were free of charge but in others, certain categories of women had to pay. This decision depended on women’s legal and residency status and in some cases health insurance. For example, Italy and Germany provided birth services for all women. In contrast, women in the Czech Republic must have a specific type of insurance otherwise they have to pay for the services.

Access to antenatal and postnatal care (Table 1) in different countries is influenced by the woman’s legal and residency status and health insurance, with women with refugee status generally having the same rights as citizens. Spain recognizes pregnant women as a vulnerable category and access to all stages of maternal care is open for women regardless of status and insurance. Some other countries (see Table 1) also consider the antenatal and postnatal periods as a condition which means women require access to maternity care services. However, in some countries, this care is not free of charge. The payment varies among countries and mostly depends on type of care, residency and legal status and insurance.

### Typology of Migration Regimes

From the 14 countries, three main types of migration regimes became apparent (Table 2). These were identified via their location in the migration context, the number of migrants in the country, gross domestic product (GDP) and household income. The types of migration regimes were linked to variables indicating public support towards migrants and their inclusion in health care (Table 3) and access to health care in general (Table 4):

1. Southern border countries of the EU are the main entry point into Europe via the Mediterranean Sea for new migrants. Within this group of countries there is a distinction between mainland countries: ‘New countries of immigration’ (Spain, Italy and Greece) and ‘Southern islands’ (Cyprus and Malta). Apart from Cyprus, the average percentage of migrants in these countries is comparable to other countries included in this group. They are categorized as high-income countries and have a positive approach toward migration, especially Italy and Spain with supportive health care policies. Greece demonstrates the lowest level of public support towards migrants and ranks as the least supportive of all included countries. Cyprus and Malta rank in the middle for public support and health care policies.
2. Post-colonial and ‘guest worker’ systems (found in the UK, Germany, Netherlands, Denmark, Norway and Switzerland) experience migration patterns established following the 2nd World War. These countries continue to be popular target countries for EU and non-EU migrants, resulting in an increased share of migrants in the country. They are high income countries with Germany and the UK having the highest economic performance. The Netherlands, UK and Switzerland have more supportive policies for migrants than Germany, Denmark and Norway. Despite this the UK has the lowest level of public support towards migrants of these countries. Switzerland has the highest share of migrants within the population and the highest level of public support towards migrants.
3. The rejecting countries (Croatia, Hungary and the Czech Republic) are East and Central European countries with a negative attitude towards migration and (excluding the Southern Islands and Greece) the lowest economic performance. These countries accept the lowest number of migrants out of the selected countries. Health care provision in the Czech Republic and Hungary is limited and not integrated into other supporting services. Croatia has more supportive health care policies.

### Migration Regime and Migrants’ Access to Standard Maternal Care

We found a relationship between a country’s health care policies, migration regime and public support towards migrants. Countries with a low index score are more likely to have policies which support maternity care for non-citizens (Table 5). However, a few countries do not show this relationship; Germany, Denmark and Norway have more positive approaches toward migration than other countries but have restrictive health care policies.

The differences between the national policies towards migrant healthcare and public support for migration could not systematically explain the variation in migrants’ access to maternity care. However, patterns emerged by types of countries, on the integration of data from Tables 1 and 5. These are presented in Table 6 below.

**Table 6:** Mapping countries by inclusion of migrants to maternity care

		Public support towards migrants and policy towards migrant healthcare	
		INCLUSIVE	LIMITED
Migrants’ access to standard maternity care	INCLUSIVE	Switzerland, Norway, Spain, Italy, UK	Malta, Croatia
	LIMITED	Denmark, Germany, the Netherlands	Cyprus, Greece, Hungary, Czech Republic

Source: own analysis

Integrating the results presented in the earlier tables thus allowed an exploration of the intersection between public support towards migrants, policy towards migrant healthcare, and access to standard maternity care granted to migrants. Access to standard maternity care to non-citizen migrants following this analysis, could be categorized into two types of countries.

First, countries which demonstrate a good fit between policies and public opinion

1. Very limited: countries with the lowest public support for migration and least flexible policy towards migrant healthcare tend to have limited migrant access to maternity care (Cyprus, Greece, Hungary, Czech Republic) in those countries, policies seem to be coherent and in line with public expectations, producing a negative outcome in relation to migrants’ rights to health care.
2. Very inclusive: countries with medium and high public support for migrants and health policy support for migrants tend to grant migrants inclusive access to standard maternity care (Switzerland, Italy, Spain, Norway, UK).

Second group: countries with lack of fit between policies and public opinion

1. Inclusive: in Malta and Croatia with medium level public support and relatively limited health policy towards migrants, migrants’ access to standard maternity care is inclusive. This suggests that pregnant migrant women can be granted elementary access to standard care even if this is unlikely to fit the contextual setting, but might be driven by international agreements.
2. Limited: in the Netherlands, with strong public and health policy support for migrants, access to standard maternity care is nevertheless limited, as it is in countries, like Germany and Denmark, where public and policy values contrast. These countries have high public support but lower health policy support for migrants and therefore migrants’ access to maternity care is limited. In these cases, restrictive policies conflict with high levels of public support towards migrants.
  While we have not explored any further factors behind these trends, this approach provides more insight than mapping migrants’ access to standard maternity care by the migration regimes only (as explored in Table 2), since in each type of migration regime there are some countries with inclusive public or health policy support to migrants, or with granting access to standard maternity care. Thus, the migration regime does not predict the level of maternity care provided, but instead it appears to be strongly dependent on everyday policy considerations with a rather low level of path dependency (e.g. Bali et al., 2022).

## Discussion

The results of the study clearly show the inconsistency in legislation around migration and maternity care across Europe despite international agreements on the rights of migrants (The European Convention on Human Rights, 1950; Convention Relating to the Status of Refugees, 1951) and high-level international commitments to universal, equitable healthcare for all (De Vito et al., 2016; WHO, 2018b). These inconsistencies and the resultant inequality in the healthcare provision for individuals, based on their legal definition as migrants, resonate with Diderichsen’s model (Diderichsen et al., 2001, 2012). They highlight the relevance of social position and social mechanisms on health outcome inequalities. Our study found refugees to be the only group of migrants who had access to standard care during pregnancy and childbirth in all countries surveyed, based on legal status alone. All other categories of non-citizen women had some restrictions on their access to maternity care mostly dependent on legal status, residency, and health insurance.

This study demonstrates that within health care systems in Europe, intrapartum care is commonly defined as an acute or emergency situation and therefore is offered to all migrant women. However, some women are charged for services regardless of their ability to pay. In addition, our findings suggest that this universal access in different countries does not cover all perinatal care. Previous research has demonstrated the importance of antenatal and postnatal care for the safety of women who are socially marginalised (e.g., Downe et al., 2009; Jones et al., 2022), yet this crucial care and effective access to maternity care is not being offered consistently or universally to a group of women many of whom are at risk of poor maternal outcomes (Heslehurst et al., 2018; Gieles et al., 2019; Fair et al., 2020).

To explore some potential explanations for the differences we found across Europe, we created a country typology. This typology sought to identify a relationship between a country’s health care policies, their migration regime and public support for migration, and explored if this was related to the policies of access to maternity care. This further analysis demonstrated significant differences within migration regimes, but also highlighted that some countries have a good fit between access policies and public support while others do not, pointing to the relative irrelevance of path dependency in these policies. This suggests that regardless of the migration regime, there is the potential to prepare a legal framework that would secure pregnant migrant women access to standard maternity care. Further research is needed to identify more precisely what other factors influence this lack of fit between the level of inclusiveness of health care policy and the level of public support towards migrants.

The period of data collection for this study was 2015–2018 which coincided with the so-called migration crisis in Europe when there were unprecedented numbers of refugees in recent times as a result of the war in Syria. The data collection for this study ended in 2018. More recently, European countries have seen migrants including refugees from new locations, including Afghanistan and, since 2022, Ukraine. There appears to be a different attitude towards Ukraine refugees with policies treating them more favourably as fellow Europeans compared to people fleeing other countries (Tozer, 2023).

It is thus important to locate further analysis with an understanding of the historical and political context within which policies are enacted, as well as considering the origin and background of a migrant population, as suggested in the earlier concept analysis of pregnant migrant women (Balaam et al., 2017). This study has explored the access to maternity care policies within different countries, however there may be a discrepancy between what policies say migrant women are entitled to and the realities of what women can access at the ground level. Further research is needed to explore the possible disjuncture between official policy and the lived experiences of migrant women within European healthcare systems. Finally, it is crucial to work with and include the voices of these (migrant) mothers in future research.

Furthermore, we would like to highlight the potential of our methodology, which allows us to consider whether migrants would have access to the standard of care in each country rather than simply comparing them to some universal and ideal standard. In the current political climate enabling “anti-gender” sentiment, our methodology may become a relevant tool in the debate about future limiting or relaxing access to reproductive care in general, not only among groups of migrants.

## Conclusions

This study documents inequities and inconsistencies in policies across European countries from 2015 to 18 that affect non-citizen migrant women’s access to maternity care. It provides a new methodological approach and a template for future analysis of policies and migration regimes/public attitudes to migration that can be applied to the changing situation within Europe. We consider the focus on access policy for maternity care as the first important step in understanding the situation faced by migrant women and working towards improving maternal care for migrant women. We include in our analysis many structural and individual factors that influence the experience and effect of care (discrimination and translation), at a policy level. Policies which affect women’s access to maternal care act to reproduce structural inequalities which compromise the health of marginalised and vulnerable women and babies in host countries. The lack of public support for migrants, leading to limited maternity access policies - or to negative experiences of care - is of concern. While it is still unclear how the more inclusive access policies emerge, it is clear from this research that there is less path dependency in this process, since migration regimes alone are not able to explain the policy outcomes. We can conclude there is space for (and likely experience with) policy innovation that is not so much dependent on the country’s policies and strategies so far but rather reflects the ability to change and innovate, sometimes also based on external pressures. These could include pressure from the EU or global bodies, migration flows, or extraordinary situations such as with health pandemics that affects access and puts pressure on everyone’s rights to care being followed.

## Data Availability

Not applicable.

## Footnotes

1 We identified a vast variety of how migrants are categorised in countries. The only consistent terms used were refugee, and asylum seeker, as an adjunct to the category refugee. This is perhaps due to these two categories having internationally accepted legal meanings based on the Geneva Convention (Convention Relating to the Status of Refugees 1951).
